# Inflammation-related biomarkers in major psychiatric disorders: a cross-disorder assessment of reproducibility and specificity in 43 meta-analyses

**DOI:** 10.1038/s41398-019-0570-y

**Published:** 2019-09-18

**Authors:** Ning Yuan, Yu Chen, Yan Xia, Jiacheng Dai, Chunyu Liu

**Affiliations:** 1 Department of Psychiatry, The Second Xiangya Hospital; Mental health Institute of the Second Xiangya Hospital; National Clinical Research Center on Mental Disorders; National Technology Institute on Mental Disorders, Central South University, Changsha, Hunan China; 2 Department of Psychiatry, Hunan Provincial Brain Hospital; Clinical Research Center for Mental Behavioral Disorder in Hunan Province, Clinical Medical School of Hunan University of Chinese Medicine, Changsha, Hunan China; 3 Center for Medical Genetics & Hunan Key Laboratory of Medical Genetics, School of Life Sciences, Central South University, Changsha, Hunan China; 4 Department of Psychiatry, SUNY Upstate Medical University, Syracuse, NY USA; 5 School of Psychology, Shaanxi Normal University, Xi’an, Shaanxi China

**Keywords:** Molecular neuroscience, Psychiatric disorders

## Abstract

Inflammation is a natural defence response of the immune system against environmental insult, stress and injury, but hyper- and hypo-inflammatory responses can trigger diseases. Accumulating evidence suggests that inflammation is involved in multiple psychiatric disorders. Using inflammation-related factors as biomarkers of psychiatric disorders requires the proof of reproducibility and specificity of the changes in different disorders, which remains to be established. We performed a cross-disorder study by systematically evaluating the meta-analysis results of inflammation-related factors in eight major psychiatric disorders, including schizophrenia (SCZ), bipolar disorder (BD), autism spectrum disorder (ASD), major depression disorder (MDD), post-trauma stress disorder (PTSD), sleeping disorder (SD), obsessive–compulsive disorder (OCD) and suicide. A total of 43 meta-analyses involving 704 publications on 44 inflammation-related factors were included in the study. We calculated the effect size and statistical power for every inflammation-related factor in each disorder. Our analyses showed that well-powered case–control studies provided more consistent results than underpowered studies when one factor was meta-analysed by different researchers. After removing underpowered studies, 30 of the 44 inflammation-related factors showed significant alterations in at least one disorder based on well-powered meta-analyses. Eleven of them changed in patients of more than two disorders when compared with the controls. A few inflammation-related factors showed unique changes in specific disorders (e.g., IL-4 increased in BD, decreased in suicide, but had no change in MDD, ASD, PTSD and SCZ). MDD had the largest number of changes while SD has the least. Clustering analysis showed that closely related disorders share similar patterns of inflammatory changes, as genome-wide genetic studies have found. According to the effect size obtained from the meta-analyses, 13 inflammation-related factors would need <50 cases and 50 controls to achieve 80% power to show significant differences (*p* < 0.0016) between patients and controls. Changes in different states of MDD, SCZ or BD were also observed in various comparisons. Studies comparing first-episode SCZ to controls may have more reproducible findings than those comparing pre- and post-treatment results. Longitudinal, system-wide studies of inflammation regulation that can differentiate trait- and state-specific changes will be needed to establish valuable biomarkers.

## Introduction

Inflammation is a regulated process that occurs in response to injury or stress. Inflammation is also implicated in psychiatric disorders. Triggered by various internal and external stressors, inflammation is moderated by the immune system through a series of signalling pathways, particularly proteins like pro- and anti-inflammatory factors. Changes of some inflammation-related factors (IRFs) have been intensively studied and reviewed in relation to psychiatric disorders^1–9^. Furthermore, pharmacology studies suggest that inflammation may have a causal effect in the development of psychiatric disorders. On one hand, drugs that target IRFs have been proposed for treating psychiatric disorders, including major depression (MDD)^10–12^, schizophrenia (SCZ)^13^ and bipolar disorder (BD)^14^, particularly in a subset of patients with chronic inflammation^10^; a recent meta-analysis of anti-cytokine treatment showed significant antidepressant effects^10^. On the other hand, some current psychotropic drugs, including antipsychotics and antidepressants, have anti-inflammatory effects, though with inconsistent findings^15–19^.

The relevance of inflammation to psychiatric disorders suggests its potential as a suitable biomarker. A biomarker is defined as “any substance, structure, or process that can be measured in the body,” which can “influence or predict the incidence of outcome or disease”^20^ as well as the treatment response^21^. Measurable in peripheral blood, IRFs have the advantage of accessibility. Currently, diagnosis of functional psychiatric disorders is based mainly on subjective interview. The addition of trait biomarkers associated with disease diagnoses can only improve the accuracy of diagnosis. Furthermore, state markers associated with specific symptoms or stages of disease can help to monitor treatment. Accordingly, it is crucial to explore whether IRFs can be used as trait and/or state markers of psychiatric disorders. IRF changes need to be associated with diseases, symptoms or treatment effects, and more importantly, such associations must be specific and reproducible to turn IRFs into biomarkers. Although dozens of meta-analyses have been published on IRFs in psychiatric disorders, the reproducibility of those results has not been thoroughly evaluated. Without comparing the studies from different disorders, the specificity of IRFs cannot be determined.

Here, we systematically reviewed meta-analysis studies of IRF changes in major psychiatric disorders. Through the evaluation, we found that meta-analyses with sufficient statistical power produced stable results. After filtering out the underpowered data, we identified IRF changes that were shared across disorders or unique to specific disorders as potential trait markers. We identified 30 IRFs showing alterations in at least one disorder. We also identified IRFs as potential state markers for specific types of patients with MDD, SCZ or BD in various comparisons. We discussed the missing aspects of existing studies and recommended future study designs needed to translate IRFs into biomarkers.

## Systematic review of meta-analyses on inflammation-related factors in psychiatric disorders

Hundreds of studies have been published on IRFs in psychiatric disorders in recent decades. Most of these studies have small sample sizes and offer limited statistical power, but statistics from these small studies can be pooled in meta-analysis, boosting power to detect true signals. Meta-analysis detects consistent changes across multiple studies. Biological or technical noises, errors and mistakes in individual studies are likely to be random and less likely affect the meta-analysis results. We chose to analyse meta-analysis publications to avoid much of the noise. We searched meta-analysis studies of IRFs in PubMed, Springer and Web of Science with the following search builder: ((((cytokine) OR ((chemokine) OR (Interleukin) OR (interferon) OR (tumour necrosis factor) OR (colony-stimulating factor) OR (growth factor) OR inflammat%))) AND (#DISORDER#) AND meta-analysis. #DISORDER# is one of the major psychiatric disorders: SCZ, BD, autism spectrum disorder (ASD), MDD, post-traumatic stress disorder (PTSD), anxiety, suicide, sleeping disorder (SD) or obsessive–compulsive disorder (OCD). For growth factors, we selected only those known to regulate inflammation, such as IGF^22^, VEGF^23^, NGF^22^, FGF^24^ and TNF^24^. The inclusion criteria were the following: 1) published in English before March 2018; 2) assessed circulating cytokine/chemokine/inflammation-related factor levels in plasma or serum samples or the central nervous system; 3) meta-analysis only. The studies involving patients of different treatment stages or symptom states (state-related) were analysed separately from the case–control studies (trait-related). We discarded two studies testing for the correlation between the IRF levels with depressive symptoms^25–27^ since their measurements were not compatible with the other differential studies. The literature selection followed the standard by Preferred Reporting Items for Systematic Reviews and Meta-Analyses (PRISMA)^28^ (Supplementary Figure, SF1).

We collected 43 meta-analysis publications on 44 IRFs in eight major psychiatric disorders. Data from 43 meta-analysis studies included SCZ (number of studies, *n* = 11), MDD (*n* = 16), BD (*n* = 6), OCD (*n* = 1), suicide (*n* = 2), ASD (*n* = 1), PTSD (*n* = 1), SD (*n* = 1), and cross-disorder studies (*n* = 4). Anxiety did not meet all our criteria for inclusion. One publication can contain meta-analyses of several IRFs in multiple disorders targeting different comparisons; therefore, we partitioned the data to serve two different analyses: trait marker analysis and state marker analysis (Table 1). Trait marker analysis targets IRF changes in undifferentiated case–control comparisons. State marker analysis targets IRF changes in patients with the same disorder but different clinical states. When a meta-analysis used patients with a clearly defined subtype or stage, like depressed, manic, acute, chronic or medicated, the study was classified into the state marker group. Otherwise, the analysis was classified as the undifferentiated case–control comparison. In those 43 meta-analyses, there were 24 trait marker studies, 6 state marker studies and 13 studies containing both trait and state analyses (Table 1). We summarised the results from the meta-analysis, including disorders and IRFs studied, sample sizes, tissue sources, statistical practice (controls for the batch effect, covariate and multiple testing inflation) and major findings (details in Supplementary Table S1). Most data were generated from peripheral blood without differentiating whole blood, serum and plasma; one study involved three IRFs in the brain^29^, and four meta-analyses were analysed in both blood and the cerebrospinal fluid (CSF)^13,29–31^. The most frequently studied disorders are MDD, BD, suicide and SCZ. These four disorders were meta-analysed numerous times, giving us an opportunity to assess the stability of the meta-analysis results over time.

**Table 1:** Use of the meta-analysis publications for trait and state marker analyses

	Trait marker analysis							State marker analysis																		
	Undifferential case–control studies							Specific-type patients–controls													Different-state patients					
								BD			MDD					SCZ					BD (diff state)			BT–AT*		
PMID	MDD	BD	BD/MDD	SCZ	PTSD	SD	Suicide	Ds	Es	Ms	Ai	Ci	BT	Me	UM	Ai	Ci	FEP	Me	UM	M–E	D–E	D–M	BD	MDD	SCZ
18005941				1																						
19375171				1																						
20015486	1																									
21641581				1												1		1								1
21872339	1																									
22687336	1																									
23419545		1																						1		
23428789				1																						
23768870		1																								
24704219				1																						
24934179																										
25529895				1																						
25541493							1																			
25678163							1																			
25897228	1																									
26065825	1																									
26123242	1																									
26140821						1																				
26169573	1																								1	
26169974				1																						1
26210676	1																									
26544749					1																					
26825882	1	1	1																							
26903140	1																									
27214525		1						1	1	1																
27898323	1			1						1				1	1		1	1	1	1						
28070123				1																						1
28122130	1																									
28338954	1	1		1																						
29088880				1																						
29133955	1																									
21796103																									1	
22336436																									1	
22749156								1	1	1											1	1	1			
24200418																										1
26903267								1	1	1	1	1				1	1	1							1	1
27838212								1	1	1											1	1				
28285148																										1
28445690													1												1	
28612257									1	1															1	
25742201								1																		
**Sum**	14	5	1	11	1	1	2	5	5	6	1	1	1	1	1	2	2	3	1	1	2	2	1	1	6	6

*BT–AT* before treatment–after treatment comparision, *Ds* depressed-state patients–controls study, *Es* euthymic-state patients–controls study, *Ms* manic-state patients–controls study, *Ai* acutely ill patients–controls, *Ci* chronically ill patients–controls, *BT* before-treatment patients–controls study, *Me* medicated patients–controls, *UM* unmedicated patients –controls, *FEP* first-episode psychosis–control, *M–E* manic patients–euthymic patients; *D–E* depressed patients–euthymic patients, *D–M* depressed patients–manic patients

### Evaluation of the stability of findings by consistency in IRFs meta-analysed multiple times

To evaluate the reproducibility of published changes of IRFs in psychiatric disorders, we examined the stability of the reported changes in IRFs that were analysed in one of the four frequently studied disorders. Ideally, reproducibility should be measured by using replicates from independent samples. Independence of samples was problematic in this study because meta-analysis studies normally use most if not all available individual studies, including those used by previous meta-analysis studies. Therefore, the different meta-analysis studies of the same IRF-disease combinations cannot be considered independent replicates. We solved this problem by using stability as a proxy of reproducibility. Even though stability is not a true measure of reproducibility, we assume that stable findings are more likely to be reproducible than unstable ones. Through the stability evaluation, we learned that studies with high statistical power (>80%) produced relatively stable findings. Therefore, we used statistical power to filter all meta-analyses and identified inflammatory changes that are likely to be reproducible.

By using the stability of the results to assess the reproducibility for the IRFs changed in BD, SCZ, suicide and MDD, we found that some findings in these meta-analyses were stable while others were not. For example, sIL-2R and sTNF-R1 were significantly elevated consistently in BD^32–34^. Some significant IRF changes only appeared in recent studies as more data became available. For example, the difference of IFN-gamma was not significant between MDD and controls in a study of 238 subjects^35^, but a later meta-analysis that includes a total of 1470 subjects found IFN-gamma to be significantly decreased in MDD patients^36^. IL-8 was reported to have no significant changes in MDD by a study of 560 subjects^37^, but it was found to be increased in MDD in a later study of 3788 subjects^38^. These conflicting results suggest that those changes can only be detected in larger sample sizes.

We hypothesised that the inconsistent results of the same IRF within the same disorder may result from underpowered studies. To test this hypothesis, we extracted the effect size measured as a standardised mean difference of every IRF in each disorder from those meta-analyses, and calculated their statistical powers by using G*power^39^ targeting *p* < 0.05. Totally, 26% of the meta-analyses did not have enough power (<80%).

For the IRF meta-analysed multiple times, 42% (16/38) of the tests showed inconsistent results in case–control comparisons. Totally, 67% (24/36) of the tests showed inconsistent results in state-related meta-analyses. After filtering out the tests with insufficient statistical power, only 16% (5/31) of case–control-related tests still showed inconsistent results. Totally, 48% (15/31 total, 4/10 of BD, 5/6 of MDD and 6/15 of SCZ) of the state-related tests remained inconsistent. Filter by statistical power clearly improved the consistency of the results by about 20% or more for both trait and state-related analyses. If two studies were completely independent, as each result has three possible states: increase, decrease and no change, the chance of two results having the same type of state is 11% (1/3 × 1/3). The observed consistency here (>33%) is much higher than 11% even in the underpowered meta-analyses. This high consistency can be partially caused by the relatedness of data used in the two meta-analyses. But the overall pattern of consistency, particularly that improved consistency associated with filter by statistical power is very encouraging. All consistencies are higher than 50% after filtering. Case–control trait-related findings have 84% consistency. The results of SCZ and BD case–control comparisons showed 100% consistency. Trait-related findings are generally more reliable than state-related tests, which have consistencies ranging between 16% and 60%.

We looked into the inclusion and exclusion criteria of all the meta-analyses and noticed large variations: 37% of the meta-analyses required diagnosis with DSM/ICD; 20% of the studies required specific age ranges; 70% of the studies removed data without controls; only 20% of the studies removed duplicated data; 11% of the studies excluded participants with other comorbidities. Very few used identical criteria. The inconsistent use of inclusion/exclusion criteria could potentially increase the inconsistencies for the results of meta-analyses. But our analyses showed sufficient consistency for those well-powered data (84% consistency for the results of case–control comparisons). The inconsistent inclusion/exclusion criteria used by different studies did not destroy the findings.

## Trait-related biomarkers

### Reproducible findings in meta-analyses from well-powered studies

Based on the stability evaluation, we decided to analyse only the data from the well-powered studies because they were more likely to yield reproducible findings. We removed the data with statistical power lower than 0.8. We applied this filter to all data, assuming that all data including those tested only once will have better reproducibility. The single meta-analysis of OCD was filtered out because of the insufficient statistical power. Thirty-eight IRFs survived the filtering. Thirty of the 38 IRFs showed significant alterations in at least one disorder (Table 2). All well-powered studies of IRFs (power > 0.8) are detailed in Supplementary Table S2.

**Table 2:** Well-powered significant changes of inflammation-related factors in psychiatric disorders and estimates of sample size required to achieve 80% power

Factor	Direction of change	Tissue	Consistency in multiple studies	Medium effect size	Largest sample size	80% Power- required sample (*p* = 0.05)	80% Power- required sample (*p* = 0.05/30, 0.0016)^a^	Effect size based on reference (PubMed ID)
*Major depressive disorder (patients vs. controls)*								
CCL11	Increase	Blood	–	0.44	454	136	340	29133955
CCL2	Increase	Blood	Y	0.96	4688	30	76	28122130 and 29133955
CCL4	Decrease	Blood	–	−0.31	507	260	680	29133955
CRP	Increase	Blood	Y	0.75	1908	48	122	26065825 and 26169573
CXCL4	Increase	Blood	–	1.03	816	26	68	29133955
CXCL7	Increase	Blood	–	0.63	771	68	170	29133955
IFN-gamma	Decrease/no change	Blood	N	−0.48	1470	114	288	28122130 and 20015486
IGF-1	Increase	Blood	–	−0.48	327	114	288	26825882
IL-10	Increase/no change	Blood	N	0.38	1283	182	454	28122130 and 22687336
IL-12	Increase	Blood	–	1.23	436	20	48	28122130
IL-13	Increase	Blood	–	1.84	616	10	26	28122130
IL-18	Increase	Blood	–	1.72	278	12	28	28122130
IL-1RA	Increase	Blood	–	0.45	258	130	326	28122130
IL-6	Increase	Blood	Y	0.62	9145	70	174	20015486, 21872339, 22687336, 26065825, 26169573, 28122130 and 28338954
IL-8	Increase/no change	Blood	N	0.42	3788	142	374	20015486, 26903140, 28122130, 28338954 and 29133955
NGF	Decrease	Blood	Y	−0.55	1010	88	220	27898323 and 25897228
sIL-2R	Increase	Blood	Y	0.47	880	120	300	21872339 and 28122130
sTNF-R2	Increase	Blood	–	1.17	195	20	54	28122130
TNF-alpha	Increase/no change	Blood	N	0.96	3077	30	76	28122130, 20015486, 21872339, 26065825 and 26169573
VEGF	Increase	Blood	Y	0.39	1754	172	432	26123242 and 26210676
*Autism (patients vs. controls)*								
CCL11	Increase	Blood	–	0.32	244	244	638	24934179
IFN-gamma	Increase	Blood	–	1.04	335	26	66	24934179
IL-1beta	Increase	Blood	–	0.65	494	60	160	24934179
IL-6	Increase	Blood	–	0.38	835	174	454	24934179
IL-8	Increase	Blood	–	0.46	290	120	312	24934179
CCL2	Increase	Blood	–	0.26	386	386	964	24934179
TGF-beta	Decrease	Blood	–	−1.06	251	24	64	24934179
*Bipolar disorder (patients vs. controls)*								
IGF-1	Increase	Blood	–	0.53	315	60	160	26825882
IL-4	Increase	Blood	Y	1.01	825	28	70	23419545 and 23768870
KYNA	Increase	Cerebrospinal fluid	–	0.79	109	42	100	28338954
NT3	Increase	Blood	–	0.38	468	174	454	27214525
NT4/5	Increase	Blood	–	0.53	401	90	236	27214525
sIL-2R	Increase	Blood	Y	0.57	616	82	206	23419545 and 23768870
sIL-6R	Increase	Blood	Y	0.49	354	110	276	23419545 and 23768870
sTNF-R1	Increase	Blood	Y	0.62	798	66	174	23768870 and 23419545
TNF-alpha	Increase	Blood	Y	0.61	985	68	180	23768870 and 23419546
*Schizophrenia (patients vs. controls)*								
CRP	Increase	Blood	Y	0.53	82,971	94	236	29088880, 23428789 and 26169974
IL-12	Increase	Blood	–	0.98	191	30	74	21641581
IL-1beta	Increase	Cerebrospinal fluid	–	0.75	102	48	122	28338954
IL-1RA	Increase	Blood	Y	0.5	371	106	266	21641581 and 18005941
IL-6	Increase	Blood	Y	1.3345	1219	18	42	18005941 and 24704219
IL-6	Increase	Cerebrospinal fluid	–	0.4	424	164	410	28338954
IL-8	Increase	Cerebrospinal fluid	–	0.35	213	213	534	28338954
KYN	Increase	Cerebrospinal fluid	–	1.22	152	20	50	28338954
KYNA	Increase	Cerebrospinal fluid	–	0.59	289	74	192	28338954
NGF	Decrease	Blood	Y	−0.633	1693	68	170	28070123 and 27898323
S100	Increase	Blood	–	2.07	738	8	22	19375171
sIL-2R	Increase	Blood	Y	0.969	1126	30	74	24704219 and 18005941
*Suicide (patients vs. controls)*								
CRP	Increase	Blood	–	0.45	456	106	326	25541493
IL-10	Increase	Blood	–	0.53	128	94	236	25541493
IL-2	Decrease	Blood	–	−0.63	133	68	170	25678163
IL-4	Decrease	Blood	–	−0.78	108	46	112	25678163
IL-6	Increase/no change	Blood	N	0.30	353	278	726	25678163 and 25541494
TGF-beta	Increase	Blood	Y	0.67	238	68	150	25678163 and 25541493
*Post-traumatic stress disorder (patients vs. controls)*								
IFN-gamma	Increase	Blood	–	0.49	158	110	276	26544749
IL-1beta	Increase	Blood	–	1.42	542	14	38	26544749
IL-6	Increase	Blood	–	0.88	1013	34	90	26544749
*Sleep disorder (patients vs. controls)*								
CRP	Increase	Blood	–	0.12	34,943	1800	4502	26140821
IL-6	Increase	Blood	–	0.20	3339	650	1624	26140821

^a^Assuming that 30 factors will be studied in one disorder, the *p* value should reach 0.05/30 to claim significance. “–” refers to single study without consistency check

We also examined the impact of tissue types on the reproducibility of findings. The single study on IRFs from the brain was under-powered^29^. Five IRFs were significantly changed in SCZ patients based on CSF samples. IL-6 was consistent with the changes detected in blood while IL-1beta was inconsistent. Three others (KYN, IL-8 and KYNA) were not studied in blood meta-analyses.

### Disease specificity based on cross-disorder comparison

To evaluate the specificity of each IRF in disorders, we investigated whether the change of one IRF occurs universally in all psychiatric disorders or only in some disorders by using only well-powered data. Whenever an IRF only shows a change in one or several disorders but not every disorder, we consider that it merits disease specificity. An IRF change is considered to be non-specific when all the disorders carry the same change.

We used the IRFs that changed for multiple disorders patients compared with controls to assess their degree of specificity. Thirteen of them were analysed in more than half (4) the disorders, including TGF-beta, IL-10, IL-1RA, IL-1beta, IL-2, IL-4, IL-6, IL-8, sIL-2R, sIL-6R, TNF-alpha, IFN-gamma and C-reactive protein (CRP). All these IRFs changed in some disorders but had no change or even opposite changes in other disorders. Among them, IL-2 is uniquely significantly low in suicide; IL-4 is significantly low in suicide, but high in BD; sIL-6R is uniquely significantly high in BD; sIL-2R is uniquely not changed in PTSD. IL-6 and CRP are the two most commonly increased IRFs changed in four disorders. The most commonly decreased IRF is the nerve growth factor (NGF) in MDD and SCZ.

**Fig. 1:**
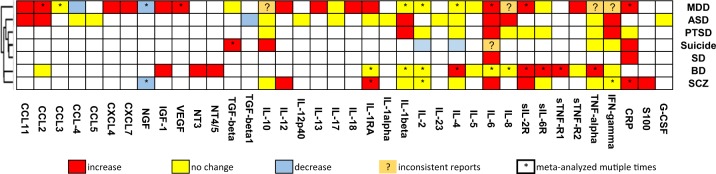
Clustering of disorders based on alterations of 38 inflammation-related factors identified from sufficiently powered meta-analyses in blood samples. Red cells are the increased changes in disorders (effect size (ES) > 0, *P* < 0.05 and power > 0.8); blue cells are the decreased changes in disorders (ES < 0, *P* < 0.05 and power > 0.8); yellow cells are the not significant changes in disorders (*P* > 0.05, power > 0.8); white cells represent missing data, either not being studied or only an underpowered study in the meta-analyses; orange cells with question marks are those with inconsistent results. “*” in the cells indicates that the significant changes are found in more than two meta-analyses. MDD, ASD, PTSD, suicide, SD, BD and SCZ have 16, 7, 3, 5, 2, 8 and 7 IRF changes, respectively

The specificity of IRFs across disorders can reflect a similarity in the aetiology or pathology of disorders. Unsupervised clustering analysis on the presence of the changes of the 30 IRFs in the seven disorders showed an interesting pattern—BD and SCZ were closer to each other than the others, while suicide clustered with PTSD and SD (Fig. 1). This pattern matched the clinical similarity among these disorders and their genetic similarity, based on published genetic studies^40^.

## State-related biomarkers

IRFs that change at different states of disorders were also examined in the well-powered studies. Two types of studies have been used in state-related analyses, including ten studies that compared BD, SCZ and MDD patients at different clinical states with controls, and twelve studies that compared pre- and post-treatment patients. Quantitative differences in IRFs among states of diseases were observed.

Out of the sufficiently powered meta-analyses, the SCZ data have the same consistency (9/15, 60%) as BD (6/10, 60%) data, and are better than MDD (1/6, 17%) data when IRFs were meta-analysed multiple times. The pre- and post-treatment comparison is one of the major sources of inconsistency (3/6 in SCZ and 5/5 in MDD). For example, IL-6 has inconsistent findings in SCZ pre- and post-treatment comparison^41,42^, but was reported to have consistent changes in several different states of SCZ: higher in acutely ill, chronically ill and first-episode SCZ patients than in controls. CRP is the only IRF that was reported to change in all three BD (manic, depressive and euthymic) states. But the depression state has conflicting results^43,44^. Studies comparing patients with first-episode SCZ patients with controls contain the most consistent results as reported in the two meta-analyses by Miller and collaborators^13,45^, many were also replicated by Upthegrove et al.^46^ except for IL-4 and IFN-gamma.

The state-related data carry more uncertainty than trait-related data. We are particularly concerned by the frequent inconsistencies observed in the pre- and post-treatment comparison studies. Among these state-related studies, findings from first-episode SCZ studies are likely more reliable. All the results are shown in Supplementary Table S3 for BD, S4 for MDD and S5 for SCZ.

It should be noted that our study was built upon meta-analysis results, which represented the consensus of multiple individual studies, less influenced than original individual studies by biological or technical noises or variations from individual studies. At the same time, meta-analyses cannot recover signals that may be missed by original studies due to technical limitations. Meta-analyses can be misled by systematic bias in individual data. Therefore, systematic investigation with better technologies and better experimental design has the potential to uncover IRF changes that we missed, or to disapprove a finding claimed here.

## Better study design to translate inflammation-related factors to biomarkers

More research is necessary before IRFs can be used as clinically useful biomarkers. We noticed some major pieces missing in previous studies, mostly due to the limitations of technology and costs. Most of those studies focused on candidate genes, which do not provide system-wide information. Statistical procedures were commonly liberal, leaving more space for false findings. Since longitudinal studies are still rare, we can neither differentiate acute and chronic changes nor can we discern trait and state markers. Lastly, blood–brain comparisons may not be necessary for developing biomarkers, but such comparisons are important for establishing the mechanisms of psychiatric disorders.

### Unbiased system-wide study

Candidate gene studies contributed to answering specific questions in biology, but they frequently missed the big picture, without assessing the impact of the genes that changed the most. Candidate genes are generally not the top signals in genome-wide or system-wide studies. The bigger differences between cases and controls with better disease specificity are hidden in the systems and remain to be discovered. Therefore, the current candidate genes, including all the IRFs reviewed here, may not be the best genes for biomarker development. The other genes discovered from system-wide hunting may be better biomarker candidates.

The regulatory system that modulates inflammation remains poorly understood. Regulation of inflammatory responses occurs at various levels including genetic, transcriptional, translational, protein modification and protein interaction, and involves many signalling pathways^47^. One GWAS of inflammation quantitative trait locus (QTL) identified some potential genetic regulators of inflammation^48^. The omics-based approaches can help reveal more about these regulatory processes. Genomic and epigenetic methods have been applied to adipose tissue^49^ and blood leucocytes^50^, leading to the discovery of large inflammatory response networks. These studies have only scratched the surface of this complex regulatory system. More remains to be learned regarding the regulation of inflammation in response to both acute and chronic stressors.

Hundreds of genes or proteins are involved in immunity and inflammation regulation and should be studied in relation to psychiatric disorders. Only a limited number of them have been investigated in psychiatric disorders due to the constraints of methodology and resources. Previous studies were completed by using the enzyme-linked immunosorbant assay (ELISA) or multiplex protein assays. Multiplex protein assay is a technology getting more attention recently^51^. It has been used in studies of alcoholism^52^, depression^53^ and autism^54^, with up to 41 IRFs examined simultaneously^52,53^. The rapidly evolving mass spectrometry-based proteomics will soon provide more comprehensive coverage. The advanced technology will make system-wide studies of inflammation feasible in the near future.

### Mandatory power analysis

Our analyses indicate that well-powered studies yield more reproducible results. Few studies reported statistical power. We performed power analysis^39^ on the 427 meta-analyses of 44 IRFs done in the eight disorders, 318 of them (74%) have sufficient power ( ≥ 0.8), while the rest were under-powered. Sample size, one of the major drivers of statistical power, is a limiting factor for many studies. The good news from this study is that the sample size required to detect the differences in IRFs between patients and controls might be relatively small for many IRFs. Based on the median effect size summarised in Table 2, if we want to test the 30 IRFs identified in this study at the same time in one disorder, the significance level can be set to *p* < 0.0016. At that *p* value, 13 IRFs would only need <100 subjects (50 cases and 50 controls) to achieve 80% power to detect a significant difference (Table 2). Certainly, since many of the IRFs have limited studies, their effect sizes in this table may be inflated. Independent testing will be needed.

### Practicing strict experimental, analytical procedures and statistical criteria

Strict quality control procedures and sufficient attention to covariance are critical towards reducing false-positive rates. From our review, we noticed that few existing studies paid sufficient attention to data quality control or statistical procedures controlling for covariates. Most IRF studies neither evaluate technical variances for their assays, nor did they report data-missing rates. These experimental factors can impact data quality. Standard requirements should be developed as common practice for IRF studies as they are for most genomic studies.

Covariates of many types should be considered. ELISA and the multiplex assay results can be easily confounded by technical artefacts, including batch effects (differences in the same sample measured at different times) and site effects (data collected from different machines may not be measured consistently). Ignoring the covariates for inflammation such as blood counts, BMI, sex and chronic stress levels can lead to false findings. The inappropriate management of covariates can misdirect meta-analyses. As shown in Supplementary Table S1, 93% of the previous studies did not address controlling batch effects for large sample sizes. Nearly 40% of the studies did not address covariates in their analyses. Collecting data from as many variables as possible and controlling them in analyses should be standard practice in future research.

Strict statistical criteria for defining significance should be consistently applied across studies. Many publications considered *p* ≤ 0.05 to be significant even when multiple IRFs were tested. With that, publication bias for the positive results likely occurred^55^. In Supplementary Table S1, about 75% of the studies did not correct for multiple testing inflation. Genetic studies of candidate genes have produced a large number of false positives in small-sample studies due to loose statistical criteria. Some scientists propose changing the threshold of significance to a smaller and more debatable value like 0.01. Inflammation studies should consider implementing a stricter threshold to reduce the number of false positives. Small-sample studies are valuable to scientific literature as they benefit meta-analysis and large consortium efforts, particularly since collecting clinical data is very challenging and very expensive. But standardised common practices to ensure high-quality data are critical for these small studies too.

### Longitudinal study for acute and chronic changes, trait and state biomarkers

Acute and chronic inflammation can have very different impacts and consequences in aetiology and pathology. Many physical and psychological stressors, environmental insults including acute smoking^56^, acute ethanol intoxication^57^, long-term smoking^58,59^, binge drinking^60^, psychological stress^61–63^, diet^64^, air pollution^65^ and infection all can induce inflammation. The inflammation levels likely also fluctuate temporally due to environmental and circadian rhythm changes regardless of health status. Single-time- point measures could be misleading. Therefore, longitudinal studies with multiple time-point measures of inflammation in peripheral tissues are necessary to accurately assess the inflammatory changes. The economical and convenient methods of data collection need to be developed for longitudinal studies.

Biomarkers will be more informative if they can differentiate traits and states to facilitate diagnosis and differential diagnosis and monitor prognosis. In this study, we only had data to compare eight psychiatric disorders. Changes of IRFs are not limited to neuropsychiatric disorders; they are also reported in patients with cardiovascular and metabolic diseases^66^, as well as cancer^67,68^. Therefore, much remains to be learned about the specificity of each individual IRF as related to various psychiatric and non-psychiatric diseases.

State markers represent the status of clinical manifestations in patients^69^. The clinical symptoms can change through the disease course while the diagnosis remains unchanged. To differentiate trait and state markers, we will need to examine patients of different episodes, illness or symptom stages, instead of lumping all patients into one case group.

A few IRFs were presented as potential state and trait markers. Our consistency analysis based on sufficiently powered meta-analyses suggests that trait-related findings might be more reliable than state-related findings so far. The state-related analyses have more inconsistent results than the trait-related analyses. More work remains to be done to accurately identify the state-related changes.

### Peripheral vs. central nervous system

Biomarkers of psychiatric disorders need to have biological relevance based on solid mechanistic understanding that will ultimately link blood and brain function. In our data, the difference of IL-12 levels in CSF between SCZ and controls was not significant^13,46^; however, IL-12 significantly increased in the blood of patients with SCZ^13,45^. We can reasonably hypothesise that inflammation could be tissue-specific. Tissue specificity may differentiate some disorders. The central nervous system may have a different inflammatory response than the peripheral tissue depending on the source of the insult. This may be particularly true in relation to the blood–brain barrier, which separates the central nervous system from peripheral circulation and responds to inflammation under its own regulation^70,71^. A systematic evaluation of the similarity and dissimilarity of inflammation across tissues in different disorders is needed. Peripheral blood data are useful for developing clinically informative biomarkers, but the brain is the ultimate affected tissue of psychiatric disorders. Therefore, building the blood–brain relationship will be critical in proving the biological relevance and illustrating the biological mechanisms. Postmortem brain data are valuable, and the PsychENCODE project^72^ brought more insights into the inflammation status of brains affected by SCZ, BD and ASD. However, resolving the causal relationships in the study of inflammation using postmortem brains will be challenging. Animal and cellular models will hold great value in understanding the regulation of inflammation^73–75^.

In summary, this systematic evaluation of inflammatory changes across multiple psychiatric disorders showed us the intriguing possibility of differentiating psychiatric disorders by using inflammatory biomarkers. Moreover, inflammation may have a large enough effect size that these biomarkers could be detected in relatively small sample sizes. We propose that a system-wide longitudinal study using strict analytical procedures could render effective and useful biomarkers.

## Supplementary information

List of Supplementary Materials

Supplementary Fig.1 Flowchart of Data Selection Using the Preferred Reporting Items for Systematic Reviews and Meta-Analyses (PRISMA)

Supplementary Table S1 -original Excel

Supplementary Table S2 -original Excel

Supplementary Table S3 -original Excel

Supplementary Table S4 -original Excel

Supplementary Table S5 -original Excel
