# Supplementary material for: Inflammation-related biomarkers in major psychiatric disorders: a cross-disorder assessment of reproducibility and specificity in 43 meta-analyses
Source: Transl Psychiatry. 2019 Sep 18;9:233. doi: 10.1038/s41398-019-0570-y (PMC6751188; doi:10.1038/s41398-019-0570-y)
Supplement: Supplementary file 1 — List of Supplementary Materials [file 41398_2019_570_MOESM1_ESM.docx]

**Supplementary Figures and Tables**

Supplementary Figure SF1. Flowchart of Data Selection Using the Preferred Reporting Items for Systematic Reviews and Meta-Analyses (PRISMA)

Supplementary Figure SF1.

**Supplementary Tables:**

**Supplementary Table S1**. Meta-analyses Included in Current Study and Major Parameters of Original Studies Used in the Meta-analyses

**Supplementary Table S2**. Effect Size, Sample Size and Statistical Information of Well-Powered Meta-analyses of Individual IRFs

**Supplementary Table S3**. Significant Changes of Inflammation-related Factors in Well-powered Meta-analyses of Different States of Bipolar Disorder

**Supplementary Table S4**. Significant Changes of Inflammation-related Factors in Well-powered Studies of Different States of Major Depressive Disorder

**Supplementary Table S5**. Significant Changes of Inflammation-related Factors in Well-powered Meta-analyses of Different States of Schizophrenia
