# Supplementary material for: Inflammation-related biomarkers in major psychiatric disorders: a cross-disorder assessment of reproducibility and specificity in 43 meta-analyses
Source: Transl Psychiatry. 2019 Sep 18;9:233. doi: 10.1038/s41398-019-0570-y (PMC6751188; doi:10.1038/s41398-019-0570-y)
Supplement: Supplementary file 2 — Supplementary Fig.1 Flowchart of Data Selection Using the Preferred Reporting Items for Systematic Reviews and Meta-Analyses (PRISMA) [file 41398_2019_570_MOESM2_ESM.doc]

**sFig.1 Flowchart of Data Selection** Using the Preferred Reporting Items for Systematic Reviews and Meta-Analyses (PRISMA)

**Screening**

**Included**

**Eligibility**

**Identification**

Records identified through PUBMED searching
(n = 62)

Additional records identified through Springer and Web of Science
(n = 112)

Records after duplicates removed
(n = 93)

Records screened
(n = 93)

Records excluded

non-immune factor analysis
(n = 43)

Full-text articles assessed for eligibility
(n = 50)

Full-text articles excluded

non-meta-analysis
(n = 7)

Studies included in qualitative synthesis
(n = 43)

Studies included in quantitative synthesis
(n = 43)


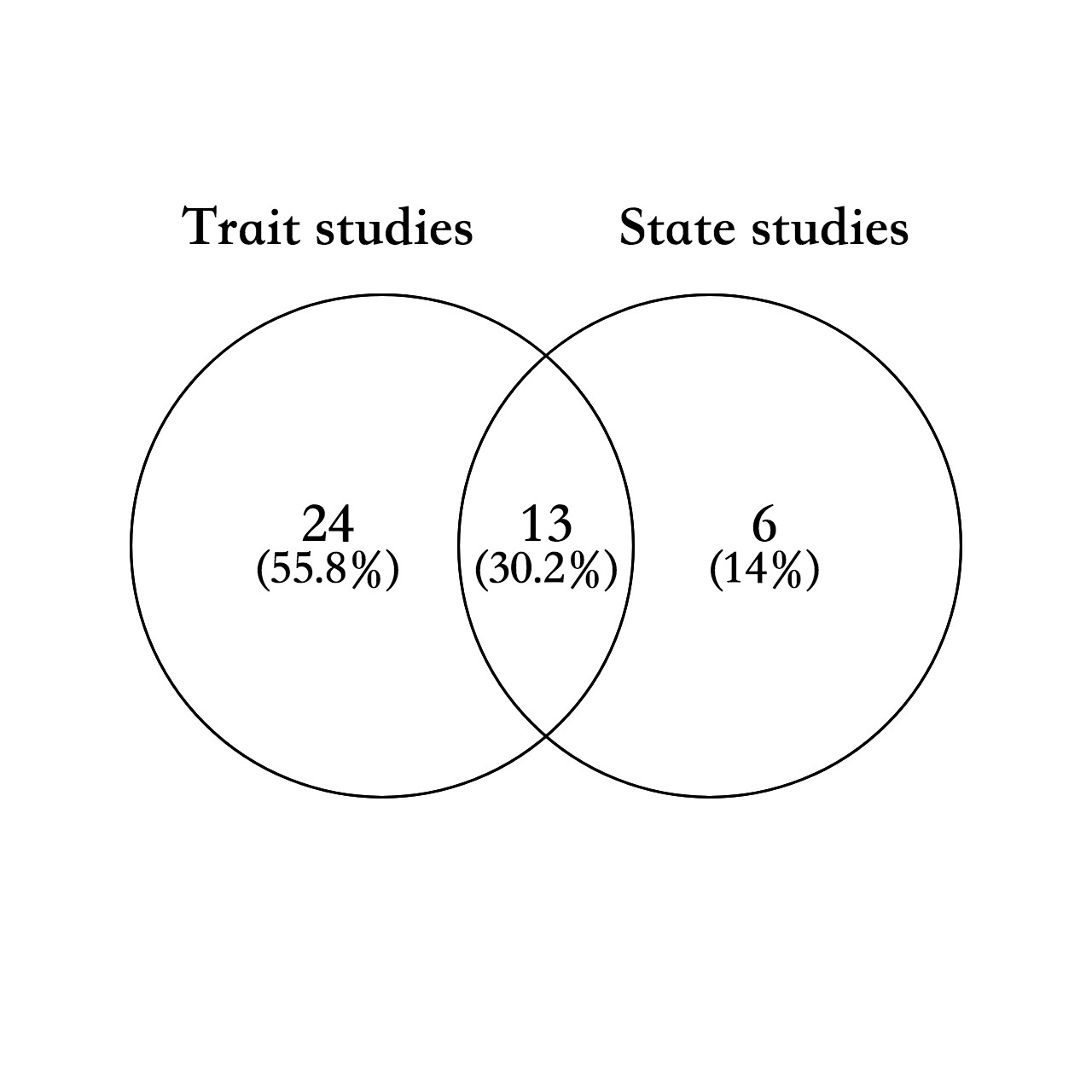


*Trait studies: comparison between undifferential patients and controls; State studies: comparison between specific state patients and controls, or comparison between specific state patients, including longitudinal studies.
